# MTA HP Repair stimulates in vitro an homogeneous calcium phosphate phase coating deposition

**DOI:** 10.4317/jced.55661

**Published:** 2019-04-01

**Authors:** María del Carmen Jiménez-Sánchez, Juan J. Segura-Egea, Aránzazu Díaz-Cuenca

**Affiliations:** 1 Department of Stomatology, Faculty of Dentistry, University of Sevilla, Sevilla, Spain; 2 Materials Science Institute of Sevilla (ICMS), Joint CSIC-University of Sevilla Center, Sevilla, Spain; 3 Networking Research Center on Bioengineering, Biomaterials and Nanomedicine (CIBER-BBN), Spain

## Abstract

**Background:**

To study the mineralization capacity *in vitro* of the bioceramic endodontic material MTA HP Repair.

**Material and Methods:**

Bioactivity evaluation *in vitro* was carried out, by soaking processed cement disk in simulated body fluid (SBF) during 168 h. The cement surface was studied by Fourier transform infrared spectroscopy (FT-IR), field emission gun scanning electron microscopy (FEG-SEM) and energy dispersive X-ray analysis (EDX). Release to the SBF media of ionic degradation products was monitored using inductively coupled plasma atomic emission spectroscopy (ICP-AES).

**Results:**

FT-IR showed increasing formation of phosphate phase bands at 1097, 960, 607 and 570 cm-1 with prolonged SBF soaking. FEG-SEM analysis reveals that HP produces a effectively surface covering consisting in homogeneous spherical phosphate phase aggregates with an average diameter of 0.5-1.0 µm. EDX analysis comparing un-treated (hydrated), 24 h and 72 h SBF treated surfaces of MTA HP Repair revealed phosphate deposition after 24 h, with high phosphorous/silicon element ratio signal measured after 24 h, indicating a very high phosphate phase deposition for this material.

**Conclusions:**

The study shows that MTA HP Repair produces a quick and effective bioactive response *in vitro* in terms of crystalline calcium phosphate surface coating formation. The high bioactive response of MTA HP Repair makes it an interesting candidate for endodontic use as repair cement.

** Key words:**Bioactive endodontic cements, bioactive response, MTA HP Repair.

## Introduction

Bioceramic endodontic cements (BECs), such as Mineral Trioxide Aggregate (MTA) and related materials, stimulate the natural remineralization process at the material-tooth interface (1). Therefore, they are considered bioactive endodontic cements (2), being applied as active therapeutic agent to stimulate regeneration (3-6).

Calcium silicates (Ca3SiO5 and Ca2SiO4) are the base compounds of BECs, together with radiopacifying additives such as bismuth, zirconia, tantalum, or tungsten oxides (2,7). Calcium silicate based cements, particularly those containing bismuth oxide as radiopacifier (8), present some disadvantages such as tooth discoloration, long setting time or difficult handling (9). Consequently, new BECs have been prepared replacing bismuth oxide with alternative radiopacifier materials (9,10).

MTA Repair HP (Angelus, Londrina, Brasil) is a new BEC in which, bismuth oxide has been replaced by calcium tungstate (CaWO4) as radiopacifier and this modification of cement composition will alter the physico-chemical characteristics and the biomechanical properties of the bioceramic material (2,11,12), and could also modify the biological functional properties (2,13,14). The aim of this study is to assess the mineralization capacity and bioactive response *in vitro* of the bioceramic endodontic cement MTA HP Repair (HP).

## Material and Methods

MTA HP Repair (Angelus, Londrina, Brasil) was used in this study. The composition of the bioceramic as the manufacturer specifications is: tricalcium silicate (Ca3SiO5), dicalcium silicate (Ca2SiO4), calcium tungstate (CaWO4) as radiopacifier, tricalcium aluminate (3CaO.Al2O3), and calcium oxide (CaO).

The *in vitro* bioactivity evaluation was assessed, by soaking the cement disks in 13 mL of simulated body fluid (SBF) (15) during 4, 24, 72 and 168 h at 36.5 oC and 60 r.p.m. shaking using polytetrafluoroethylene beakers. Previously to the bioactivity assay, the samples were sterilized under UV light for 10 min period on each side. SBF solution was filtered using 0.2 mm bacteriostatic filter (Biofil).

Fourier transform infrared (FT-IR) spectra of as-processed set material and the SBF treated samples were collected in transmission configuration in the 1300-400 cm-1 range using 4 cm-1 intervals in a Nicolet IS50 FT-IR (Thermo Scientific, Madison WI, USA). The microstructures were studied by field emission gun scanning electron microscopy (FEG-SEM) using a HITACHI S-4800 (Tokyo, Japan). Images were recorded at an accelerating voltage of 2 kV. Energy dispersive X-ray (EDX) analysis was carried out at 10 kV with an EDX Bruker XFlash 4010 detector. Concentrations of Si, Ca, P, W and Al ions in the soaking media were monitored after 72 and 168h by inductively coupled plasma atomic emission spectroscopy (ICP-AES) using the spectrometer Horiba Jobin Yvon (Ultima 2, Paris, France). Control solutions consisting of pure SBF was simultaneously prepared and stored under the same conditions.

## Results

**Figure 1:**
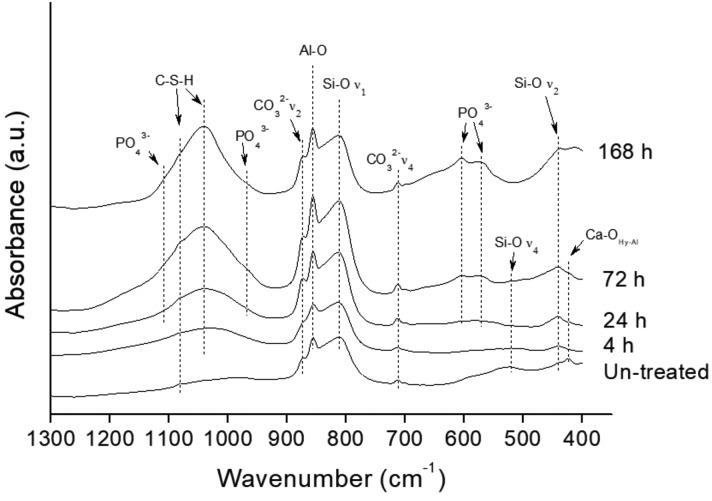
Fourier transform infrared (FT-IR) spectra of MTA HP Repair after SBF treatment of the different analysed times plotted together with the spectra of the SBF un-treated samples. (S-C-H = calcium silicate hydrate).

The FT-IR absorbance spectra of MTA HP Repair after the analysed SBF treatment times, in comparison with the spectra of the as-set (SBF un-treated) sample, are shown in Figure 1. An important intensity increase with treatment time, of calcium silicate hydrate C-S-H broad band within the 1000-1100 cm-1 range is observed. Likewise, increasing formation of phosphate phase bands at 1097, 960, 607 and 570 cm-1 (16), are clearly observed with prolonged SBF soaking. Calcium hydroxyapatite growing on the MTA HP Repair surface after 72 h treatment can be inferred from the two bands at 607 and 570 cm-1 characteristics of phosphate in a crystalline environment (17). Incipient signals at 607 and 570 cm-1 are observed at only 24 h SBF treatment.

**Figure 2:**
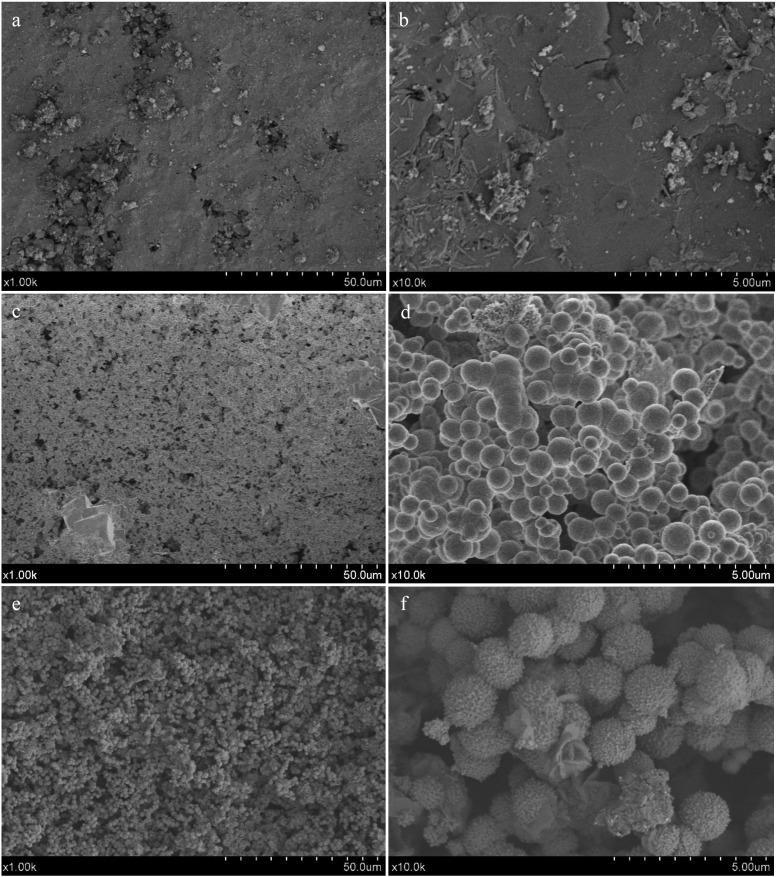
Field emission gun scanning electron microscopy (FEG-SEM) of MTA HP Repair. Back- scatter electron micrographs of un-treated surfaces (a, b); and secondary electron micrographs after 24 h (c-d) and 72 h (e-f) SBF treatment. Scale bars: (left (50 um); right (5 um)).

**Figure 3:**
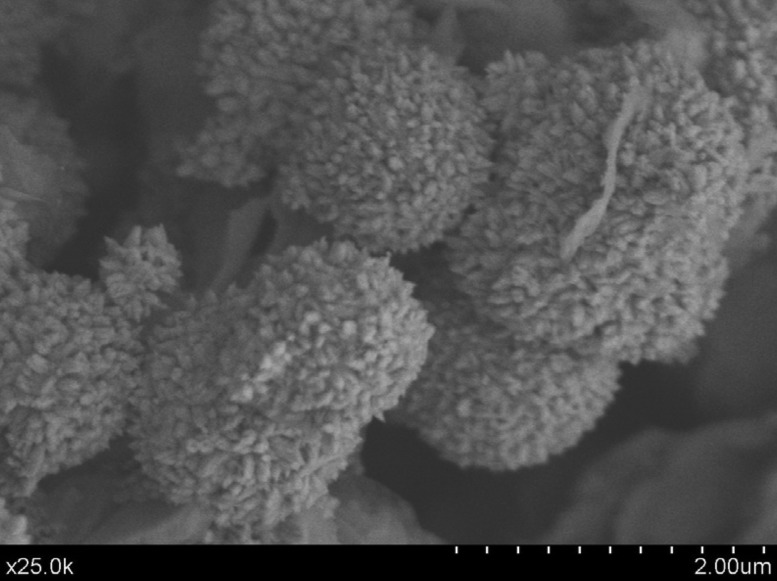
Field emission gun scanning electron microscopy (FEG-SEM) of MTA HP Repair: secondary electron micrograph after 72 h SBF treatment. Scale bar: (2 µm).

**Figure 4:**
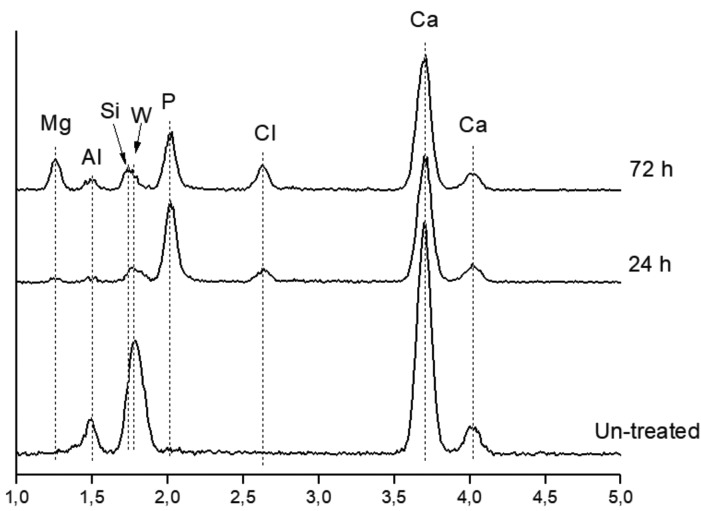
Energy dispersive X-ray (EDX) analyses of un-treated and after 24 h and 72 h SBF treatment of MTA HP Repair.

Back-scatter FEG-SEM micrographs of the un-treated MTA HP Repair set material surface (a, b), as well as secondary images after 24 h (c, d) and 72 h (e, f) SBF treatment, at two different magnifications are presented in Figure 2. New homogeneous spherical aggregate formations covering the surface of the SBF treated samples are observed after 24 h soaking time, showing average diameter spheres in the 0.5-1.0 µm range. After 72 h SBF treatment, a visible growing in size of the spherical features is observed. Besides, prismatic features characteristic of calcium apatite-like structure with hexagonal symmetry, growing out of the spherical formations are clearly visible in Figure 3. Calcium phosphate deposition is confirmed by EDX analysis. Un-treated, 24 h and 72 h SBF treated surfaces of MTA HP Repair are displayed in Figure 4. The analysis reveals the detection of a high phosphorous signal after only 24 h, indicating a very high phosphate phase deposition for this material.

Si, Ca, P, W and Al ionic product in solution after 72 and 168 h SBF soaking treatment are presented in  Table 1. Si and Ca, major BEC material components, elution indicates material dissolution. W and Al ions were also detected in solution being Al particularly high, showing up to 0.4 mg L-1 concentration after 168 h.

**Table 1:**
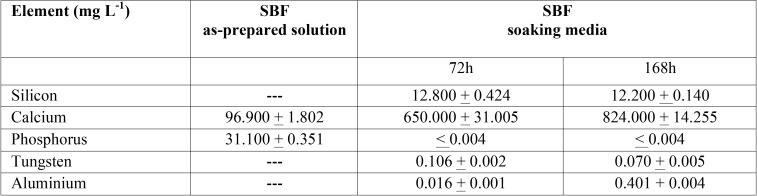
Si, Ca, P, W and Al ionic product degradation concentration in the SBF soaking media of MTA HP Repair material monitored by inductively coupled plasma atomic emission spectroscopy (ICP-AES).

## Discussion

Calcium phosphate mineralization capacity of MTA HP Repair, a bioceramic cement with calcium tungstate as radiopacifier used in endodontic therapy, has been studied. Bioactivity assay *in vitro* indicates that MTA HP Repair forms a structured crystallized calcium phosphate coating layer after 72 h treatment. FEG-SEM observations of MTA HP Repair surface after 24 h only SBF treatment, shows a phosphate phase coating formed of homogeneous spheres with an average diameter in the 0.5-1.0 µm range. This high bioactive response might be associated to the high calcium aluminate content of this material (18), and to the great surface degradation, as measured by the Si and Ca ion product release, detected by ICP. The nucleation of the calcium phosphate phase would be favoured by the change in the chemical composition and in the pH of the solution resulting from the accumulation of dissolution products from the MTA HP Repair, with the appearance of new surface sites (19). Particularly, the repolymerization of a porous silica-rich layer through silanol groups condensation from the soluble silica in the form of Si(OH)4, followed by the migration of Ca2+ and PO43- groups to the surface forms a film rich in amorphous CaO-P2O5.

The clinical manifestation of bioactivity with the use of BECs has been attributed to phosphate phase’s mineralization induction capacity (16) and compared to that of calcium hydroxide postulating that the mechanisms of action were similar (20). When MTA HP Repair is exposed to a phosphate-containing media such as SBF, a series of reactions take place on the surface between calcium from the cement and phosphate from the solution consisting of the absorption of Ca and P ions on the silica-rich (C-S-H) surface and the precipitation of HPO42- containing phase which matures into a crystalline hydroxycarbonate apatite phase at increasing treatment times. Micrograph in Figure 3 displays, resolved elongated nano-metrical grains with crystalline morphology growing out of spherical formations, which have been confirmed as calcium phosphate composition by FT-IR and EDX. We argue that, the new formed calcium phosphate layer could filled the superficial crack and voids providing a stable sealing at cement surface. Also, that phosphate ions able to promote apatite deposition, from biological fluids and blood present at the external surface during surgical procedures, could well enhance the formation of a mineralised biomimetic cement-tissue interphase. A recent investigation carried out by Qiu *et al.* (21) using MTA extract has shown that concentrations of Ca ions of 88.9 mg L-1 and Si ions of 0.22 mg L-1 in the culture medium promote the repair of injured pulp, potentially by accelerating proliferation and reducing the time required for hDPCs to enter into the odontoblastic differentiation stage in the clinical setting.

The results of the present study indicates that MTA HP Repair produces a quick and effective bioactive response *in vitro* in terms of HA surface coating formation. The high bioactivity response for HP is in good accordance with its high calcium aluminate content. Recently, a study carried out on human dental pulp stem cells has demonstrated the high biocompatibility and cytocompatibility of MTA Repair HP (7).

## Conclusions

Bioactivity essay *in vitro* indicates that MTA HP Repair produces a quick and effective bioactive response *in vitro* in terms of homogeneous calcium phosphate surface coating formation. MTA HP Repair stimulates the formation of a nanostructured calcium phosphate coating layer after 72 h treatment. The high bioactive response of MTA HP Repair makes it ideal for endodontic use as repair cement.
